# Enhanced Efficiency of flySAM by Optimization of sgRNA Parameters in *Drosophila*

**DOI:** 10.1534/g3.120.401614

**Published:** 2020-10-05

**Authors:** Decai Mao, Yu Jia, Ping Peng, Da Shen, Xingjie Ren, Ruibao Zhu, Yuhao Qiu, Yuting Han, Jinchao Yu, Qinyun Che, Yutong Li, Xinyi Lu, Lu-Ping Liu, Zhao Wang, Qingfei Liu, Jin Sun, Jian-Quan Ni

**Affiliations:** * Gene Regulatory Lab, School of Medicine, Tsinghua University, Beijing 100084, China; † Sichuan Academy of Grassland Science, Chengdu 611731, China; ‡ Tsinghua University-Peking University Joint Center for Life Sciences, Beijing 100084, China; § School of Pharmaceutical Sciences, Tsinghua University, 100084 Beijing, China; ** Shandong First Medical University & Shandong Academy of Medical Sciences, Jinan 250000, China; †† Tsingdao Advanced Research Institute, Tongji University, Qingdao 266000, China

**Keywords:** flySAM, sgRNA, position effect, GC content, DNA strand

## Abstract

The flySAM/CRISPRa system has recently emerged as a powerful tool for gain-of-function studies in *Drosophila melanogaster*. This system includes Gal4/UAS-driven dCas9 activators and U6 promoter-controlled sgRNA. Having established dCas9 activators superior to other combinations, to further enhance the efficiency of the targeting activators we systematically optimized the parameters of the sgRNA. Interestingly, the most efficient sgRNAs were found to accumulate in the region from -150bp to -450bp upstream of the transcription start site (TSS), and the activation efficiency showed a strong positive correlation with the GC content of the sgRNA targeting sequence. In addition, the target region is dominant to the GC content, as sgRNAs targeting areas beyond -600bp from the TSS lose efficiency even when containing 75% GC. Surprisingly, when comparing the activities of sgRNAs targeting to either DNA strand, sgRNAs targeting to the non-template strand outperform those complementary to the template strand, both in cells and *in vivo*. In summary, we define criteria for sgRNA design which will greatly facilitate the application of CRISPRa in gain-of-function studies.

Loss-of-function (LOF) and gain-of-function (GOF) are the conventional methods to address the genetic mechanisms behind biological processes and diseases. Although both of these two complementary approaches are important for biomedical research, LOF dominates the gene function studies in *Drosophila melanogaster* using transgenic RNAi techniques and the CRISPR/Cas9 genome editing system (Ni *et al.* 2009; Ni *et al.* 2011; Ren *et al.* 2013; Ren *et al.* 2014a; Xu *et al.* 2015; Jiao and Gao 2016; Qiao *et al.* 2018). In contrast to LOF, the method for GOF in *Drosophila* heavily depends on the traditional Gal4/UAS overexpression system (Brand and Perrimon 1993). Although this system allows tissue- or developmental stage-specific expression of genes of interest, the method is laborious and time-consuming. Therefore, it remains a major challenge in *Drosophila* to generate a GOF transgenic resource on a genome-wide scale. In an effort to overcome this challenge, a novel transcriptional activation system in *Drosophila* based on the CRISPR/Cas9 technique, termed the “CRISPR transcriptional activation (CRISPRa) system”, has been developed in recent years (Konermann *et al.* 2015; Lin *et al.* 2015; Ewen-Campen *et al.* 2017). The CRISPRa system is created by fusing the appropriate activation domains to dCas9 and targeting them to upstream of the target gene’s transcriptional start site (TSS) through specific sgRNAs. Activation domains can then recruit transcriptional factors to the promoter region and initiate transcription of the target. The specificity of the CRISPRa system is controlled by a 20bp guide sequence in sgRNA, and thus we can simply change the 20bp guide sequence to modulate different targets. This approach opens up the possibility to build a GOF transgenic library (Zirin *et al.* 2020).

Previously, we generated a flySAM-based CRISPRa system in *Drosophila*, by using a method of T2A-mediated multiple protein expression, which includes the Gal4/UAS system-controlled flySAM and U6b promoter-driven sgRNA (Jia *et al.* 2018). We have observed that the flySAM system can significantly activate target genes with only one sgRNA, and that the severity of phenotypes is comparable to the traditional Gal4/UAS overexpression system. Importantly, the flySAM system can target and modulate multiple genes simultaneously, with only one genetic cross. Therefore, the flySAM CRISPRa system provides an ideal GOF method in *Drosophila*, with the characteristics of high efficiency, high specificity and easy manipulation, paving the way to building a genome-wide GOF transgenic resource.

However, when applying the flySAM system in GOF research, we found that the activation efficiency varies dramatically when using different sgRNAs targeting the same gene, and the efficiency of the CRISPRa system particularly depends on the guide sequence in the sgRNA. This highlighted the need for a systematic evaluation of parameters affecting sgRNA activity. In the present study, we first determine the position effect of sgRNAs, and show that efficient sgRNAs accumulate in the region from -150bp to -450bp upstream of the TSS. We also show that the activation efficiency is strongly correlated with the GC content of the sgRNA targeting sequence: the higher the GC content, the more effective being the activation. In addition, we demonstrate that sgRNA placement is more important than its GC content: sgRNAs targeting areas beyond -600bp from the TSS cannot activate target genes even when containing 75% GC. Finally, we compare the activities of sgRNAs targeting different DNA strands, and surprisingly find that they have a strand preference. sgRNAs targeting to the non-template strand (NT strand) outperform those complementary to the template strand (T strand), both in cells and *in vivo*. In summary, we define criteria for sgRNA design which will greatly promote the application of CRISPRa in GOF studies.

## Materials and Methods

### Fly strains

*MS1096-Gal4* (Bloomington #8860) was used to drive expression in wing. *Ubi-Gal4* was used to perform qRT-PCR assay (Owusu-Ansah *et al.* 2008). All CRISPRa stocks were obtained from THFC and are listed in Table S1.

### Generation of CRISPRa lines

All the sgRNA sequences used in generating transgenic CRISPRa stocks are listed in Table S1. After annealing, the sgRNA was cloned into flySAM2.0 vector (Jia *et al.* 2018). Then the constructed vectors were injected into *y,sc,v,nanos-integrase;attP40* embryos to screen the transgenic lines following standard procedures (Wu *et al.* 2019).

### Phenotypic and statistics analysis

For phenotypic analysis, adult wings (10-20 per sex, per genotype) were mounted in 1:1 ethanol/glycerol and imaged using a Nikon Ti-e microscope. For statistical analysis, GraphPad Prism and Excel were used to calculate the means and s.d.

### Construction of luciferase reporter and CRISPRa plasmids

sgRNA target sites with the PAM sequence were synthesized and annealed then cloned into the *SpeI/BamHI*-digested (to remove the U6B: sgRNA elements) sgRNA2.0 luciferase reporter vector to form luciferase reporter plasmid (Jia *et al.* 2018). In these plasmids, firefly luciferase gene is under the control of the HSP70b promoter and the sgRNA target site is 255bp (for experiments comparing the effects of T and NT strand) or 53bp (for remaining experiments) upstream from the TSS.

CRISPRa plasmid was modified from the flySAM2.0 vector. The vermilion-attB-gypsy-UAS-DSCP fragment of flySAM2.0 vector was replaced with an Act5C promoter amplified from the pAc5.1A vector (forward primer: CGAATTGGGTACAAGCTTAAAATCATGAATGGCATCAACTCTG; reverse primer: TGGGGCCATGGTGGCGGTACCGTCTCTGGATTAGACGACTGCTG) using the Hieff Clone Plus Multi One Step Cloning Kit (Yeasen Biotech, Catlog#10912ES10). The resulting vector is named the CRISPRa plasmid. Like construction of CRISPRa lines, sgRNAs targeting luciferase reporter were cloned into the CRISPRa plasmid; see Table S2 for sgRNA sequences.

### Cell culture and transfection

*Drosophila* S2 cells were cultured at 25° in Schneider’s *Drosophila* Medium (Invitrogen 21720024) containing 10% FBS (PAA, A005N). Cells cultured in the 96-well plate were co-transfected with luciferase reporter plasmid and appropriate CRISPRa plasmid, using X-tremeGENE HP DNA Transfection Reagent (Roche, 06366236001) according to the manufacturer’s instructions.

### Luciferase assay

Luciferase assays were performed 48h after transfection using the Steady-Glo Luciferase Assay kit (Promega, E2520). Each sample was added to 100ul luciferase reagent. After incubation in the dark for 20min, luminescence was measured on a luminometer (Thermo Scientific, VARIOSKAN FLASH). More than three independent samples were used for each luciferase assay, and the averages were used in comparisons. For comparison between individual experiments, the value was further normalized with results obtained from samples transfected with only luciferase reporter plasmid.

### qRT-PCR

Total RNA was isolated from an entire fly in adult stage using TRIzol reagent (Thermofisher Scientific, 15596026). A total of 1.5μg RNA was used to make cDNA, using the GoldScript cDNA Kit (Invitrogen, c81401190) according to the manufacturer’s protocol. qRT-PCR was performed using SYBR Premix Ex Taq (TAKARA, RR420A) and analyzed with the iQ5 real-time PCR detection system (Bio-Rad). Results were normalized against *rp49* expression.

Primer sequences for qRT-PCR experiments are listed below:

**Table:** 

rp49-qF	TACAGGCCCAAGATCGTGAAG
rp49-qR	GACGCACTCTGTTGTCGATACC
sip3-qF	TGTTCGGCAAGCTGCTAAG
sip3-qR	GTCATCCCGGAATACGGTAAAG
bataGlu-qF	TGAACGCCAATCGCAAAGAAC
bataGlu-qR	ACAACAATCCCTTGGTTGGTG

### Data availability

The authors affirm that all data necessary for confirming the conclusions of the article are present within the article, figures, and tables. Strains are available upon request. Figure S1 is the statistical results of sgRNAs targeting the downstream of the transcriptional start site. Table S1 contains CRISPRa lines used in this study. Table S2 contains CRISPRa plasmids used in this study. Supplemental material available at figshare: https://doi.org/10.25387/g3.13047701.

## Results

### Most efficient sgRNAs target the region From -150bp to -450bp of the TSS

**Figure 1:**
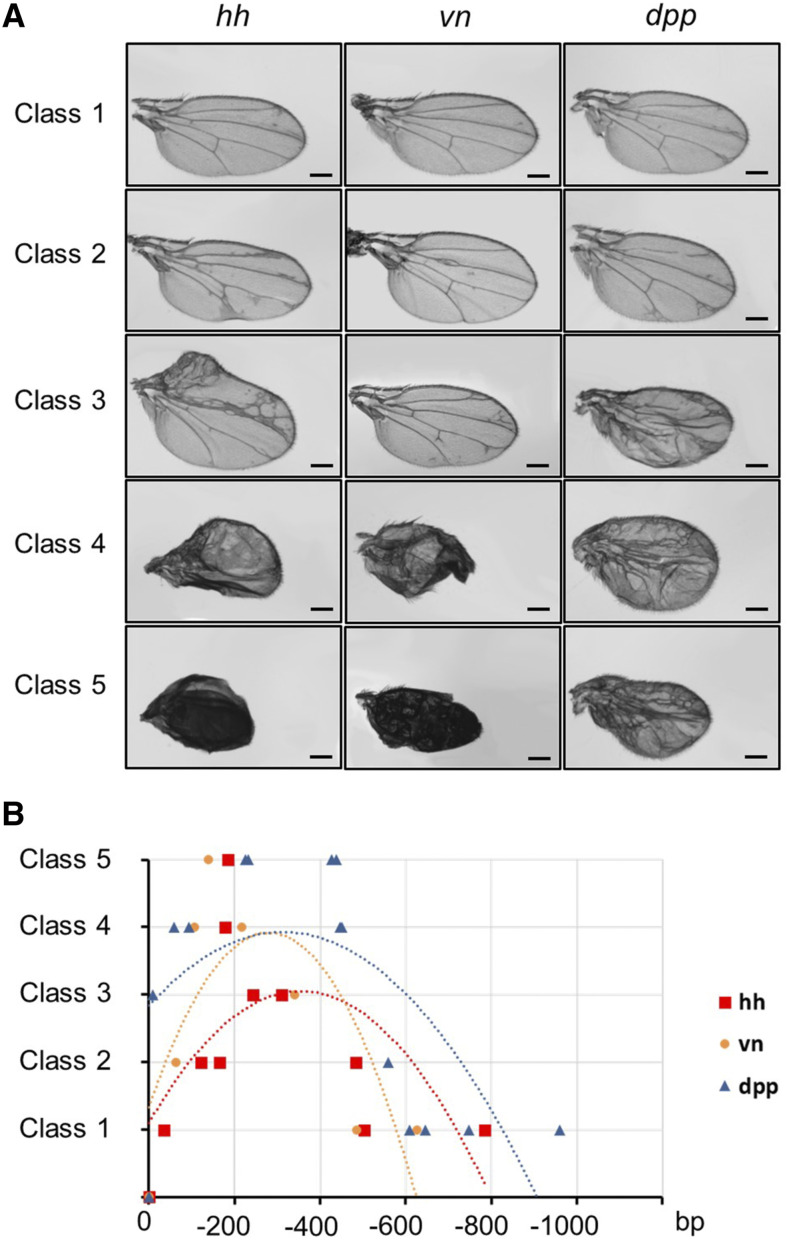
The criteria for sgRNA placement in the flySAM2.0 system. (A) Images of wings from gene activation using *MS1096-Gal4*. The wing phenotypes were divided into five classes: class 1, a few ectopic veins (*hh*), wild type (*vn*), a few ectopic veins (*dpp*); class 2, thick veins (*hh*), a few ectopic veins around L3 (*vn*), a few ectopic veins and veins disappearing (*dpp*); class 3, duplicated wing (*hh*), ectopic vein at the distal end of L2-L5 (*vn*), some ectopic veins and small (*dpp*); class 4, duplicated wing and crinkled (*hh*), moderately crinkled wing (*vn*), extensive ectopic veins and small (*dpp*); class 5, severely crinkled (*hh*), severely crinkled wing (*vn*), extensive ectopic veins and small (*dpp*). (B) Statistical analysis of the relationship between wing phenotype and sgRNA placement relative to TSS. The x-axis shows the distance from sgRNA to TSS. 11, 7, 13 sgRNAs were designed for *hh*, *vn* and *dpp*, respectively.

Having established that flySAM outperforms all other approaches (Jia *et al.* 2018), we tried to define the position effect of sgRNAs from a window between 0 and -600bp upstream of the TSS, and carried out examinations using a sensitive wing assay for sgRNAs targeting *hh*, *v*n and *dpp*. When flySAM is driven by *MS1096-Gal4*, expressed in entire wing, the activation of *hh*, *vn* and *dpp* shows dose-dependent phenotypes ranging from mild to serious wing defects, termed as class 1 to class 5 (Figure 1A). After statistical analysis, it was found that sgRNAs targeting the region from 0 to -150bp or -450 to -600bp of the TSS often generated moderate wing defects, but defects appeared mild or there was no effect if further than 600bp from the TSS. Interestingly, the most active sgRNAs targeting these three genes that give rise to class 4 or 5 phenotypes are all located in the region from -150- to -450bp (Figure 1B), and the majority accumulate around -200bp, suggesting this position as the first choice to select sgRNA to activate gene transcription. We also designed sgRNAs targeting 5′UTR, gene body and 3′UTR regions of *vn*, *dpp*, *ci* and *wg* respectively, but no severe phenotypes were observed (Figure S1). Taken together, to efficiently activate the endogenous gene, the sgRNAs should be designed in a window from -150 to -450bp of the TSS, and around -200bp is optimal, while sgRNAs will not be effective if targeting far away from TSS, *i.e.*, longer than -600bp.

### Activation efficiency is highly correlated with the GC content of sgRNAs

**Figure 2:**
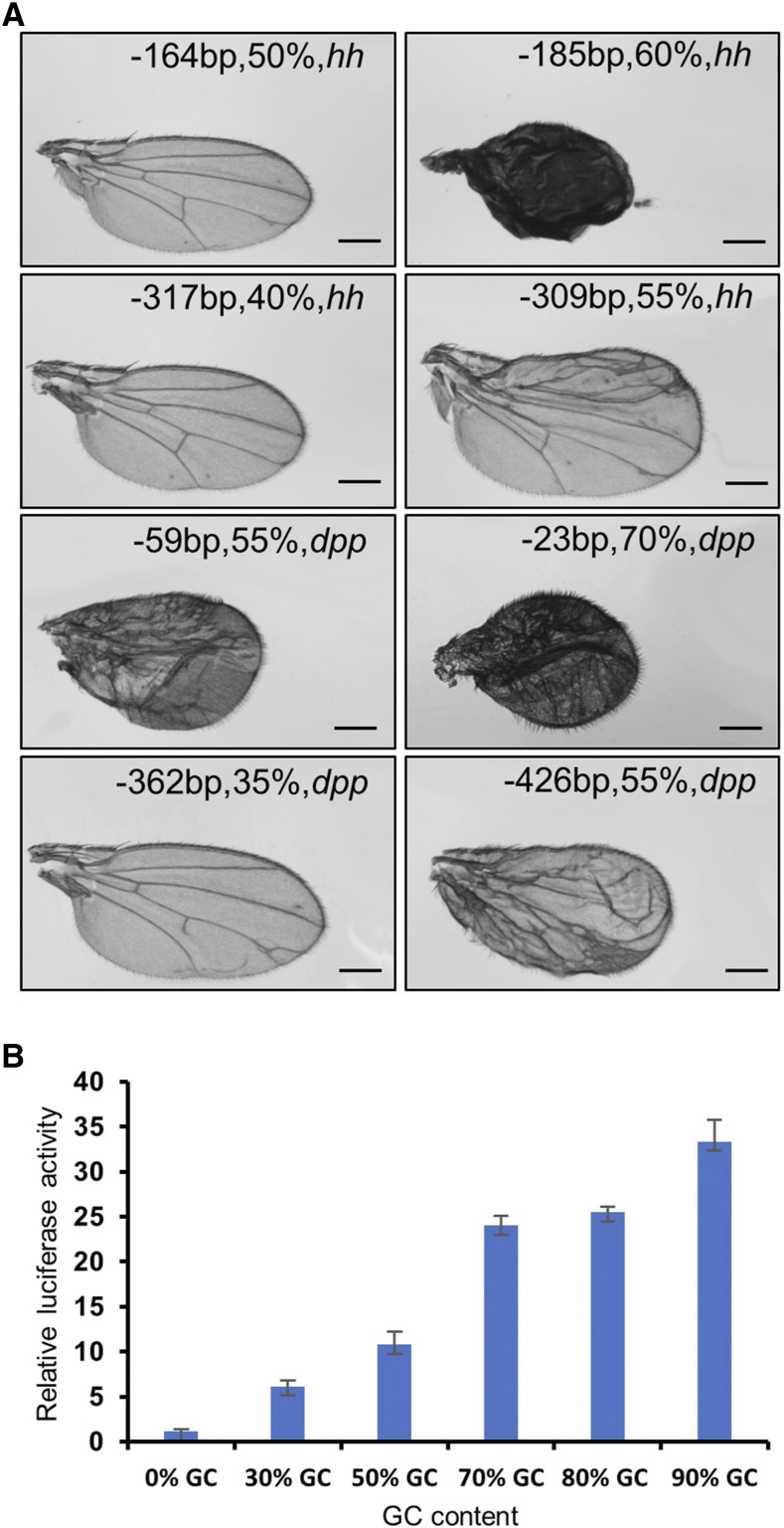
Effects of sgRNA GC content on target gene activation. (A) Wing phenotypes generated by sgRNAs with different GC content but from the same region. The label on the upper side of each wing represents the position, GC content and gene name. Two pairs of sgRNAs were designed for each gene. FlySAM was expressed in the wing using *MS1096-Gal4*. (B) Quantification of activity: six sgRNAs of varying GC content targeting the same region of the luciferase reporter construct were transfected into S2 cells (n = 3, mean ± SD).

Different sgRNAs from the same region trigger different levels of activation, most likely due to the GC content of the 20bp spacer sequence of the sgRNA, as the hydrophobic interaction within the GC base pair is stronger than that in AT. This suggests that increasing the GC content will enhance the sgRNA binding capability, thereby providing a stable platform to efficiently recruit the dcas9-activating complex. To test this possibility, we designed a series of sgRNAs from the same region but with differing GC content targeting *hh* and *dpp*, and then evaluated the efficiency of activation when these flySAM transgenic flies were driven by *MS1096-Gal4*. As we expected, sgRNAs with high GC content always generated more severe phenotypes than nearby sgRNAs with low GC content, either targeting *hh* or *dpp* (Figure 2A). To confirm the effect of GC content on the ability of a sgRNA to activate the target gene, we constructed a luciferase reporter, and systematically analyzed the performance of sgRNAs with spacer sequences of differing GC content, from 0 to 90%. In this reporter system, the firefly luciferase gene is controlled by the HSP70 basal promoter, and a series of 20bp sgRNAs targeting DNA sequences with different GC contents were designed and cloned into the same position upstream of the HSP70 promoter. After co-transfection with the different sgRNAs in CRISPRa plasmid into S2 cells, we collected the cells and measured the luminescence intensity triggered by these sgRNAs. Consistent with the phenotypic assay, activation efficiency is highly associated with the GC content of the spacer sequence: from twofold luminescence intensity of 0 GC content to 35-fold of 90% GC content (Figure 2B).

**Figure 3:**
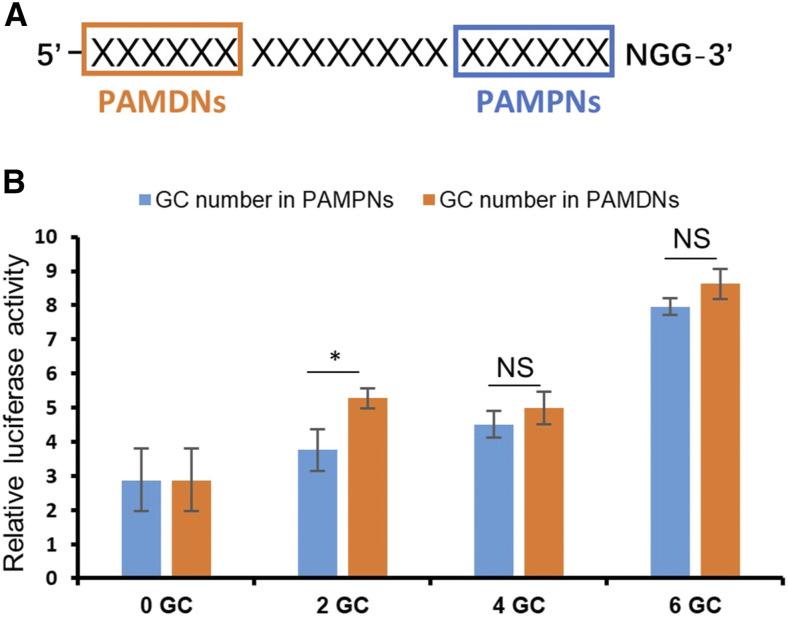
The location of GCs in sgRNAs did not affect activation efficiency. (A) Diagram of PAMDNs and PAMPNs. NGG is the PAM sequence. (B) Relative luciferase expression levels triggered by sgRNAs with different amounts of GC in PAMDNs (orange) or PAMPNs (blue). Data are evaluated with two-tailed Student’s *t*-test (*P < 0.05, n = 3, mean ± SD).

It has been previously reported that the GC content within the 6bp closest to the protospacer-adjacent motif (PAM) is critical for CRISPR-mediated DNA editing (Ren *et al.* 2014b). To determine whether this applies also to CRISPRa, we compared the effect of increasing the GC content between the 6bp of PAM-proximal nucleotides (PAMPN) and the PAM-distant nucleotides (PAMDN) (Figure 3A). According to the luminescence intensity, increasing GC number in either PAMPN or PAMDN significantly promoted the transcriptional activity to a similar level, further supporting that increasing GC content enhances transcriptional activation, but indicating no preference for the region of the spacer sequence (Figure 3B). All together, these systematic analyses suggest that effective sgRNA can be designed based on the GC content, the more GC number within the 20bp spacer sequence of the sgRNA, the higher transcriptional activation will be achieved.

### High GC content sgRNAs lost efficiency if further than 600bp from TSS

**Figure 4:**
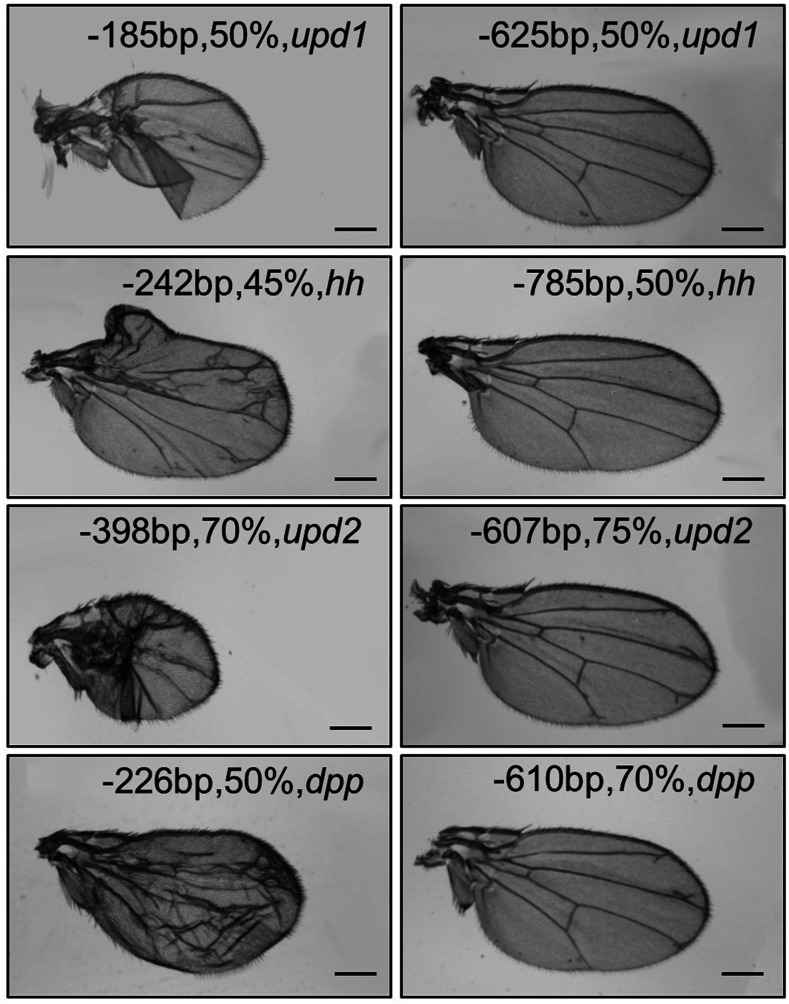
sgRNA placement is more important than GC content. Wing phenotypes were generated by sgRNAs with differing GC content and location using *MS1096-Gal4*. The label on the upper side of each wing represents the position, GC content and gene name. A pair of sgRNAs were tested for each gene, one located in the optimal window and the other targeting the region further than 600bp upstream of the TSS.

Based on the above data, using sgRNAs that exceed -600bp of the TSS significantly reduces the ability to activate gene transcription, while using sgRNAs with higher GC content can promote gene activation. We therefore considered whether increasing the GC content can compensate for the reduction of activation beyond -600bp. To test this, we designed sgRNAs targeting *upd1*, *upd2*, *hh* and *dpp*, and generated flySAM transgenic flies. Compared with the sgRNAs close to the TSS that often trigger severe phenotypes when driven by *MS1096-Gal4*, these sgRNAs targeting the region further than -600bp from the TSS all appeared to have a minor or no effect on wing development, even with GC content increased to 75% (Figure 4). This indicates that even sgRNAs with higher GC content will not trigger transcriptional activity if the distance is longer than 600bp from the TSS.

### Targeting NT strand is more efficient than T strand

**Figure 5:**
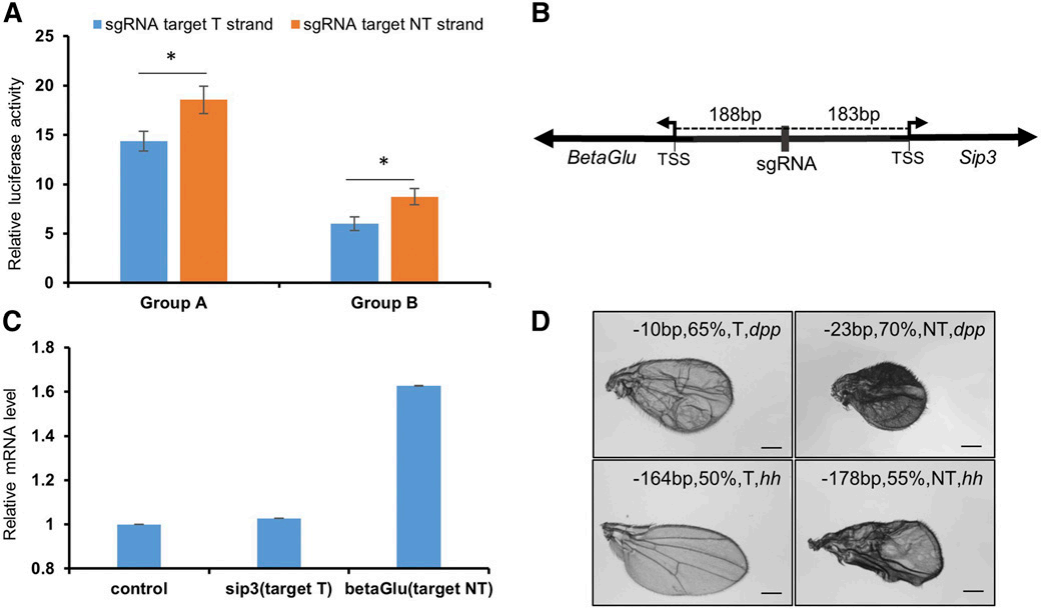
sgRNA is more efficient in targeting NT strand than T strand. (A) Quantification of sgRNA activity targeting different DNA strands (Group A and Group B). sgRNAs targeting T and NT strand in Group A or B are reverse complement. Relative luciferase expression levels of the sgRNAs targeting the NT strand were all significantly higher than the T strand. Data are evaluated with two-tailed Student’s *t*-test (*P < 0.05, n = 3, mean ± SD). (B) Diagram of a sgRNA in the middle of the *BetaGlu* and *Sip3* intergenic region. The sgRNA is complementary with the NT strand of *BetaGlu* and the T strand of *Sip3*. (C) Relative endogenous gene activation caused by the sgRNA indicated in (b). qRT-PCR results show that the sgRNA activated *BetaGlu* more efficiently than *Sip3* (n = 3, mean ± SD). (D) Phenotypic comparison of different sgRNA lines driven by *MS1096-Gal4*. sgRNAs from same region and with similar GC content targeting the NT or T strand of *dpp* and *hh* were selected. The label on the upper side of each wing represents the position, GC content, targeting strand and gene name.

In DNA transcription, the template strand (T strand) is the DNA strand whose base sequence corresponds to the base sequence of the RNA, while the non-template strand (NT strand) associates and directs RNA polymerase II transcription. A previous report suggested that there is no difference in CRISPR interference efficiency when targeting either the T or NT strand (Ghosh *et al.* 2016). To examine whether this is applicable to the flySAM system, we performed an *in-vitro* luciferase assay and *in-vivo* qRT-PCR, as well as a phenotypic assay. Surprisingly, based on two groups of sgRNAs for comparison, the luminescence intensity from the sgRNAs complementary to the NT strand was significantly higher than from those complementary to the T strand (Figure 5A). In addition, we selected two endogenous genes, *BetaGlu* and *Sip3*, where the intergenic sequence between their TSSs was less than 400bp (Figure 5B). One sgRNA targeting the middle of the intergenic region was designed to build transgenic flies in flySAM. After driving by *Ubi-Gal4*, we collected the adults and performed qRT-PCR. Consistent with the *in-vitro* assay, the sgRNA targeting the NT strand of *BetaGlu* efficiently triggered activation, while on the T strand of *Sip3* the transcripts were only slightly increased (Figure 5C). Furthermore, we selected several sgRNAs from the same region and with similar GC content targeting *hh* and *dpp*. When transgenic flies generated with these sgRNAs were driven by *MS1096- Gal4*, the phenotypes from sgRNAs targeting the NT strand were all more severe than from those targeting the T strand (Figure 5D), supporting a DNA strand bias for flySAM that sgRNAs targeting the NT strand is more efficient than those targeting the T strand.

## Discussion

With its genetic versatility and simplicity, *Drosophila* has been one of the best organisms to study developmental biology, including but not limited to neurobiology, cell biology, immunobiology and behavior. Most recently, the flySAM system has been developed, enabling us temporally and spatially to activate one or multiple endogenous genes simultaneously. The design ingeniously integrates three transcriptional activators, and has been proven more effective than other derivatives (Jia *et al.* 2018). Since this system relies on Cas9-fused activators and sgRNA to specifically and efficiently activate the target gene, we turned to look at the parameters of the sgRNA. After systematic analysis, we found that the most efficient sgRNAs accumulate in the region from -150bp to -450bp upstream of the TSS, and specifically around -200bp, and the activation efficiency is strongly positively correlated with the GC content of the 20bp spacer sequence within the sgRNA. Interestingly, the target region is critical and dominant to the GC content, as sgRNAs selected from upstream of -600bp of the TSS fail to trigger transcription even when containing 75% GC. Surprisingly, when comparing the activities of sgRNAs targeting either DNA strand, sgRNAs targeting the NT strand outperform those complementary to the T strand. Normally, the region from -150bp to -450bp is the upstream of the promoter, and belongs to one of the most important regulatory sequence to spatially and temporally control the transcription, and the region far from -600bp TSS will often lose the enhancer ability. The promoter region (<-150bp), the gene body, the 3′-UTR or the transcribed strand, either may be occupied by transcriptional factors to impede the sgRNA/dcas9-activation complex, or the association of the sgRNA/dcas9-activation complex may prevent the procedure of RNA polymerase II initiation or elongation.

**Figure 6:**
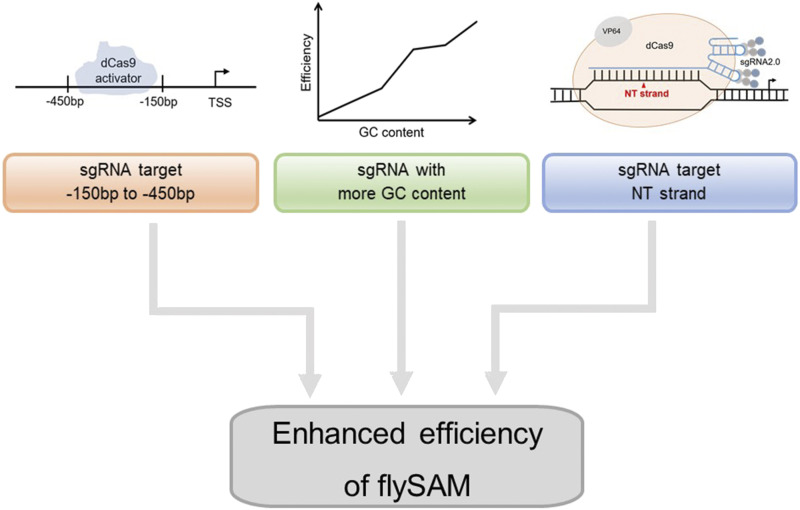
Parameters of sgRNA that affect activation efficiency. sgRNAs in the region from -150bp to -450bp upstream of the TSS (left), more GC content of the 20bp spacer sequence within the sgRNA (middle) and targeting the NT strand (right) can enhance the efficiency of flySAM.

In conclusion, considering all these parameters of sgRNA that affect activation efficiency, such as the position, GC content and DNA strand (Figure 6), we can select the best sgRNAs to improve the ability of flySAM to activate genes both in cells and *in vivo*. In addition to simple design and low cost, the flySAM system can activate all the transcripts of a target gene in gradient, modulate multiple genes simultaneously by co-expressing multiple sgRNAs in a single fly. With this transgenic fly, we can also easily combine the existing resource to perform mosaic analysis of the interaction between different cells. As for the potential limitation, when sgRNA/dcas9-activation complex is driven by some Gal4 drivers, it may have side effects due to the toxicity of dcas9. All together, the criteria we have here defined for sgRNA design will greatly improve the accessibility and worth of the flySAM activation system to the *Drosophila* community.
